# A prospective analysis of two studies that used the 5-mm interval slices and 5-mm margin-free method for ipsilateral breast tumor recurrence after breast-conserving surgery without radiotherapy

**DOI:** 10.1007/s12282-022-01406-5

**Published:** 2022-09-30

**Authors:** Shozo Ohsumi, Reiki Nishimura, Norikazu Masuda, Sadako Akashi-Tanaka, Kimito Suemasu, Hideko Yamauchi, Eriko Tokunaga, Tadashi Ikeda, Tsunehiro Nishi, Hiroto Hayashi, Yuichi Iino, Yuichi Takatsuka, Yasuo Ohashi, Hideo Inaji

**Affiliations:** 1 Department of Breast Oncology, National Hospital Organization Shikoku Cancer Center, 160 Kou, Minami-umemoto-machi, Matsuyama, Ehime 791-0280 Japan; 2 Department of Breast Oncology, Kumamoto Shinto General Hospital, Kumamoto, Kumamoto Japan; 3 Department of Breast and Endocrine Surgery, Nagoya University Graduate School of Medicine, Nagoya, Aichi Japan; 4 Department of Breast Surgical Oncology, Show University School of Medicine, Tokyo, Japan; 5 ARCHE Clinic, Saitama, Saitama Japan; 6 Department of Breast Surgical Oncology, St. Luke’s International Hospital, Tokyo, Japan; 7 Department of Breast Oncology, National Hospital Organization Kyushu Cancer Center, Fukuoka, Fukuoka Japan; 8 Department of Surgery, Kitasato University Kitasato Institute Hospital, Tokyo, Japan; 9 Toda Chuo Health Exam Center, Toda, Saitama Japan; 10 Department of Surgery, National Hospital Organization Kanmon Medical Center, Shimonoseki, Yamaguchi Japan; 11 Department of Breast and Thyroid Surgery, Kusunoki Hospital, Fujioka, Gunma Japan; 12 Department of Breast Surgery, Kansai Rosai Hospital, Amagasaki, Hyogo Japan; 13 Faculty of Science and Engineering, Chuo University, Tokyo, Japan; 14 Department of Breast Surgery, Kaizuka City Hospital, Kaizuka, Osaka Japan

**Keywords:** Breast cancer, Breast-conserving treatment, Radiotherapy, Surgical margins, Prospective study

## Abstract

**Background:**

Breast-conserving surgery with radiotherapy is one of standard treatments for early breast cancer. However, it is regarded as an option to treat elderly patients with small hormone receptor-positive breast cancer with breast-conserving surgery and hormone therapy without radiotherapy. We conducted two sequential prospective studies to examine the feasibility of breast-conserving surgery without radiotherapy since 2002 and present the results.

**Patients and methods:**

Primary female breast cancer patients who fulfilled the strict eligibility criteria were prospectively enrolled in two sequential studies named WORTH 1 and 2. The surgical materials were sliced in 5-mm intervals and all slices were examined microscopically. Postoperative radiotherapy was not allowed, but tamoxifen or anastrozole was administered for 5 years. Ipsilateral breast tumor recurrence (IBTR)-free survival was the primary outcome.

**Results:**

The data of the two studies were combined (*N* = 321). The median follow-up period for IBTR was 94 months (4–192 months). Only three patients were treated with adjuvant chemotherapy. The 5- and 10-year IBTR-free rates were 97.0% and 90.5%, respectively. The age at operation and PR status affected IBTR rates independently. When we calculated IBTR-free rates of patients who were 65 years of age or older at the time of surgery and had PR-positive tumors, the 5- and 10-year IBTR rates were both 98.4%.

**Conclusions:**

Our “5-mm-thick slice and 5-mm free-margin” method may be effective to select patients who can be treated by breast-conserving surgery and hormone therapy without radiotherapy.

## Introduction

Breast-conserving surgery with radiotherapy is one of the standard treatments for early breast cancer. There were no differences in disease-free and overall survival between breast-conserving treatment with radiotherapy and mastectomy in randomized trials and meta-analysis [1–8]. Radiotherapy was demonstrated to markedly reduce ipsilateral breast tumor recurrence (IBTR) in many randomized trials and meta-analysis [7, 9–12]. However, radiotherapy is time-consuming and costly, and can cause severe treatment-related adverse events [13].

Randomized trials have been conducted to identify the population not requiring radiotherapy after breast-conserving surgery [14]. Accordingly, it has been regarded as an option to treat elderly patients with small hormone receptor-positive breast cancer by breast-conserving surgery and hormone therapy without radiotherapy. However, the 10-year IBTR rate in patients treated without radiotherapy was around 10% in the randomized trials. As such, a good method to reduce the IBTR rate without radiotherapy is needed. We therefore conducted two sequential prospective studies to examine the feasibility of breast-conserving surgery without radiotherapy using our 5-mm interval slice and 5-mm margin-free method since 2002.

## Patients and methods

Primary female breast cancer patients who fulfilled the following eligibility criteria were prospectively enrolled in two sequential studies named WORTH 1 and 2: (1) a tumor of 3 cm or less by palpation, (2) pathologically node negative by axillary dissection or sentinel node biopsy and M0, (3) no treatment before surgery, (4) 50 years of age or older at the time of surgery and postmenopausal, (5) no tumor cells within 5 mm from the margins histologically, (6) no lymphatic invasion around the primary cancer, (7) estrogen receptor-positive judged by each institution, and (8) within 8 weeks after definitive surgery. The surgical mammary gland materials must have been sliced in 5-mm intervals and all the slices must have been examined microscopically. The status of hormone receptors and HER2 was judged at each institution. We regarded HER2 in immunohistochemistory (IHC) 3 + , or 2 + and FISH amplification as positive. But we included HER2 IHC 2 + without the information of FISH in negative in Table 1. Postoperative radiotherapy was prohibited, but adjuvant chemotherapy was allowed. The patients were administered tamoxifen or anastrozole in WORTH 1 and anastrozole in WORTH 2 as postoperative adjuvant endocrine therapy for 5 years. The exclusion criteria were: (1) suspected multifocality of the tumor, (2) history of breast cancer or bilateral breast cancer, or (3) psychological disease.

**Table 1:** Characteristics of the patients, and their tumors and treatments

	WORTH 1 (*n* = 123)	WORTH 2 (*n* = 198)	Total (*n* = 321)
Median age at operation	65 (range 51–84)	66 (range 50–84)	65 (range 50–84)
Median tumor size by palpation	1.6 cm (range 0–3.0 cm)	1.4 cm (range 0–4.0 cm)	1.5 cm (range 0–4.0 cm)
Personal history of cancer			
Yes	5 (4.2%)	19 (9.6%)	24 (7.5%)
No	118 (95.9%)	179 (90.4%)	297 (92.5%)
Axillary surgeries			
Sentinel node biopsy	75 (61.0%)	183 (92.4%)	258 (80.4%)
Axillary dissection	44 (35.8%)	8 (4.0%)	52 (16.2%)
None	3 (2.4%)	7 (3.5%)	10 (3.1%)
Unknown	1 (0.8%)	0	1 (0.3%)
Progesterone receptor status			
Positive	86 (69.9%)	171 (86.4%)	257 (80.1%)
Negative	37 (30.1%)	27 (13.6%)	64 (19.9%)
HER2 status			
Positive	2 (1.6%)	1 (0.5%)	3 (0.9%)
Negative (IHC 2 + but FISH was not done)	105 (85.4%) (5)	190 (96.0%) (3)	295 (91.9%) (8)
Unknown	16 (13.0%)	7 (3.5%)	23 (7.2%)
Histological types			
Invasive ductal cancer	102 (82.9%)	174 (87.9%)	276 (86.0%)
Ductal carcinoma in situ	10 (8.1%)	11 (5.6%)	21 (6.5%)
Others	11 (8.9%)	13 (6.6%)	24 (7.5%)
Adjuvant hormonal agents			
Tamoxifen	86 (69.9%)	15 (7.6%)	101 (31.5%)
Aromatase inhibitors	34 (27.6%)	178 (89.9%)	212 (66.0%)
Others	2 (toremifene) (1.6%)	0	2 (0.6%)
Unknown	1 (0.8%)	4 (2.0%)	5 (1.6%)
None	0	1 (0.5%)	1 (0.3%)
Adjuvant chemotherapy			
Yes	0	3 (1.5%)	3 (0.9%)
No	122 (99.2%)	192 (97.0%)	314 (97.8%)
Unknown	1 (0.8%)	3 (1.5%)	4 (1.2%)

IBTR-free survival and distant relapse-free survival (DRFS) were recorded as the interval from initial definitive surgery until IBTR or distant relapse, respectively. Only IBTR that occurred as the first event was regarded as events of IBTR-free survival. IBTR that developed at the same time as other relapses or within 2 months of the time of other relapses was regarded as an event of IBTR-free survival. Patients who did not develop IBTR or distant relapse were censored at the time of last follow-up or death as the IBTR-free rate.

Survival rates were calculated using the Kaplan–Mayer method. Statistical analyses were conducted using the log-rank test for univariate analyses or proportional hazards model for multivariate analysis. Only variables that were significant in the univariate analyses were included in the multivariate analysis. *P* values of < 0.05 were considered significant.

The primary end point was IBTR-free survival, and secondary end points were DRFS and overall survival.

As safety monitoring, interim monitoring by Bayesian approach was carried out. As stopping criteria based on the posterior distribution, WORTH 1 was planned to stop if the posterior probability exceeding 1% in the annual IBTR rate exceeded 95%. WORTH 2 adopted a criterion of 0.5% in the annual IBRT rate. The determination of the sample size for WORTH 1 can be shown with an accuracy such that the upper limit of the 95% confidence interval of the estimated IBRT rate is about 10% when the 5-year IBRT rate is assumed to be 5%. In WORTH 2, assuming that the 5-year IBRT rate is 2.5%, the accuracy that the upper limit is within about 5% is obtained. The sample size for WORTH 1 was set 120 and that for WORTH 2 was 200.

The protocols were approved by the ethics committee at each participating institution.

Written informed consent was received from all participants.

Registration of WORTH 1 was not required in 2002 when it was started. WORTH 2 was registered in UMIN-CTR, numbered UMIN000000534, on December 10, 2006.

## Results

The number of patients who participated in the two trials was 123 and 198 in WORTH 1 and WORTH 2, respectively. The characteristics of patients, and their tumors and treatments are shown in Table 1. Almost all patients were HER2 negative and others in histological types in Table 1 had invasive cancers other than invasive ductal carcinoma.

### WORTH 1 study

The patient enrollment for WORTH 1 was conducted between October 2002 and March 2005. In total, 123 patients participated in WORTH 1. The median age at the time of surgery was 65 years (range 51–84). The median tumor size by palpation was 1.6 cm (range 0–3.0 cm). The median follow-up period for IBTR was 102 months (range 18–192 months). The hormonal agents used were tamoxifen in 86 patients, anastrozole in 34, toremifene in 2, and unknown in 1. Adjuvant chemotherapy was not administered to all patients, but one lacked information about adjuvant chemotherapy.

The 5- and 10-year IBTR-free rates were 94.8% and 87.7%, respectively. The 5- and 10-year overall survival rates were 98.3% and 95.1%, respectively, and 5- and 10-year distant DRFS rates were 98.3% and 94.9%, respectively.

We examined the effects of age at the time of surgery, tumor size by palpation, and progesterone receptor (PR) status on the IBTR rates, but none significantly affected the IRTR rates, although older ages, smaller tumor sizes, and PR positivity slightly reduced IRTR rates.

### WORTH 2 study

One hundred and ninety-eight patients were enrolled in WORTH 2 between December 2006 and November 2011. The median age at the time of surgery was 66 years (range 50–84) and ages were unknown in two patients. The median tumor size by palpation was 1.4 cm (range 0–4.0 cm) and the tumor sizes were not known in 18. The median follow-up period for IBTR was 88 months (range 4–145 months). Anastrozole was used in 167 patients, exemestane in 11, tamoxifen in 15, unknown in 4, and no hormonal agent was used in 1. One hundred and ninety-two patients did not receive adjuvant chemotherapy, whereas adjuvant chemotherapy was administered to three and three lacked information.

The 5- and 8-year IBTR-free rates were 98.4% and 92.9%, respectively. The 5- and 8-year overall survival rates were 98.9% and 96.2%, respectively, and 5- and 8-year distant DRFS rates were 100% and 97.6%, respectively.

We analyzed the effects of age at the time of surgery, tumor size by palpation, and PR status on IBTR-free rates. Older patients developed IBTR significantly less frequently than younger patients (5-year IBTR-free rates: 97.6% for 64 years or younger vs. 99.0% for 65 or older, *P* = 0.044). There was no difference in IBTR between the large and small tumors (5-year IBTR-free rates: 97.4% for 1.3 cm or smaller vs. 98.9% for 1.4 cm or larger, *P* = 0.698). PR positivity significantly increased the IBTR-free rate (5-year IBTR-free rates: 99.4% for PR positive vs. 91.4% for PR negative, *P* = 0.0003).

### Results of the combined analyses

The combined data of WORTH 1 and 2 are presented hereafter. The median age at the time of surgery was 65 years (range 50–84). The median tumor size by palpation was 1.5 cm (0–4.0 cm). The median follow-up period for IBTR was 94 months (4–192 months). Only three patients received adjuvant chemotherapy.

**Fig. 1:**
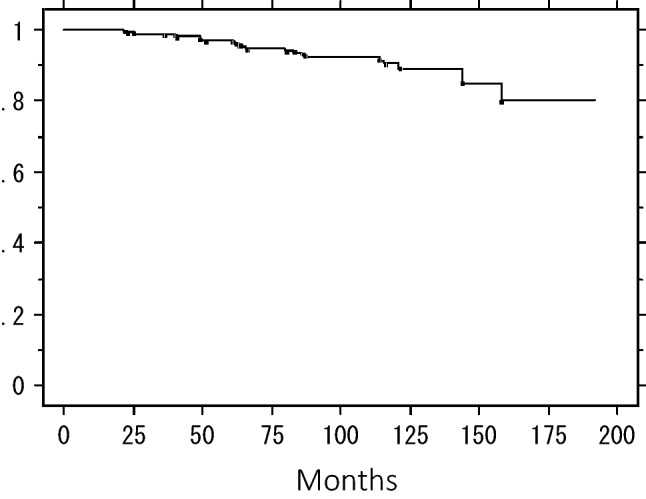
Ipsilateral breast tumor recurrence-free rate in the combined patients

**Fig. 2:**
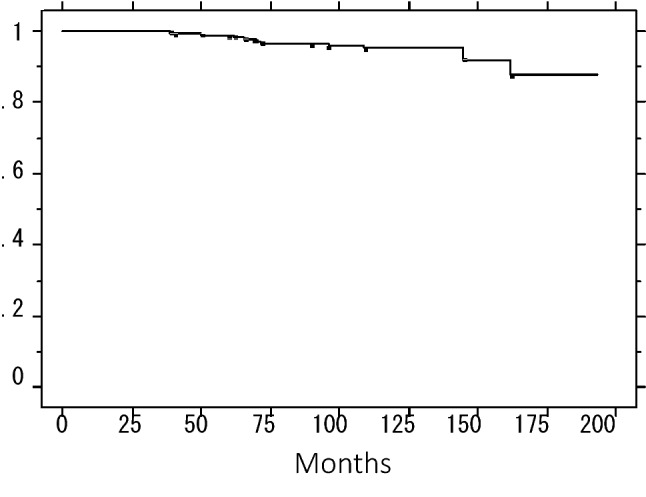
Overall survival of the combined patients

**Fig. 3:**
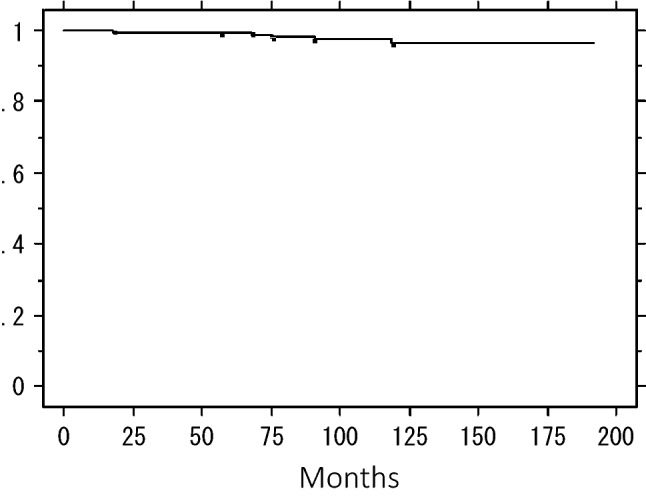
Distant disease-free survival of the combined patients

The 5- and 10-year IBTR-free rates were 97.0% and 90.5%, respectively (Fig. 1). The 5- and 10-year overall survival rates were 98.7% and 95.1%, respectively (Fig. 2), and 5- and 10-year distant DRFS rates were 99.3% and 96.3%, respectively (Fig. 3).

**Fig. 4:**
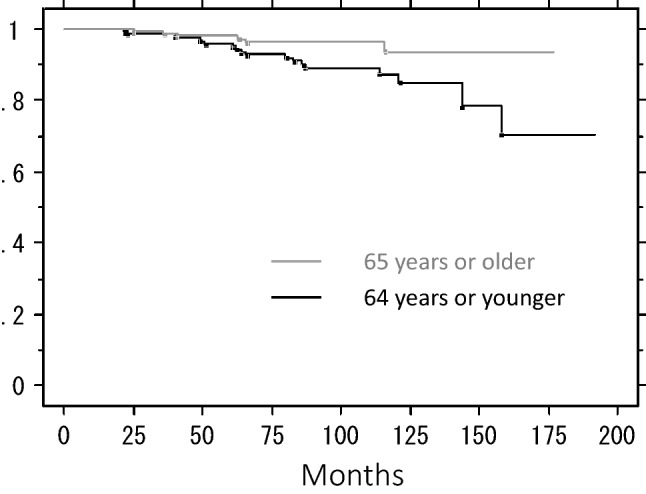
Ipsilateral breast tumor recurrence-free rate by age at the time of surgery

**Fig. 5:**
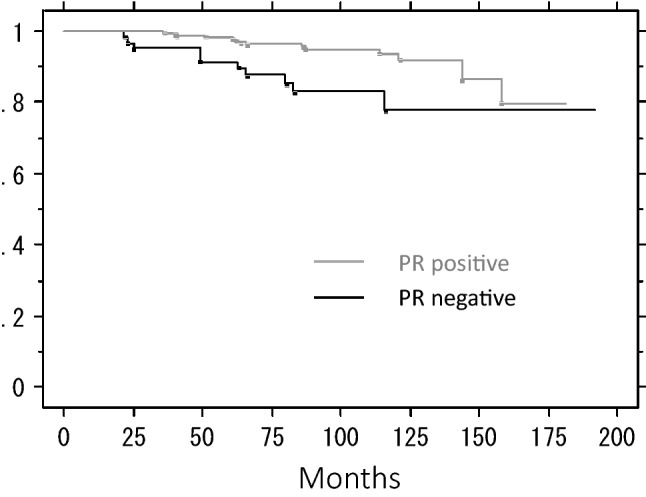
Ipsilateral breast tumor recurrence-free rate by PR status

We analyzed the effects of age at the time of surgery, tumor size by palpation, PR status, and hormonal agents on IBTR-free rates. Older patients developed IBTR significantly less frequently (5-year IBTR-free rates: 95.8% for 64 or younger vs. 98.1% for 65 or older, *P* = 0.025) (Fig. 4). There was no significant difference in the IBTR-free rate between the patients with large and small tumors (5-year IBTR-free rates: 96.9% for 1.4 cm or smaller vs. 96.8% for 1.5 cm or larger, *P* = 0.125). PR positivity significantly increased the IBTR-free rate (5-year IBTR-free rates: 98.3% for PR-positive vs. 91.5% for PR-negative, *P* = 0.006) (Fig. 5). Hormonal agents had no effect (5-year IBTR-free rates: 95.9% for tamoxifen or toremifene vs. 97.4% for aromatase inhibitors, *P* = 0.435). Both the tumor size and PR status affected IBTR-free rates independently (64 years or younger, *P* = 0.024, hazard ratio 2.90: 95% confidence interval 1.15–7.31) (PR-negative, *P* = 0.007, hazard ratio 3.10: 95% confidence interval 1.37–6.99). When we calculated the IBTR-free rates of patients aged 65 years or older at the time of surgery with PR-positive tumors (*N* = 136) in the combined population in WORTH 1 and 2, the 5- and 10-year rates were both 98.4%.

## Discussion

Breast-conserving surgery with radiotherapy to the breast is one of the standard local treatments for early breast cancer because randomized trials and meta-analyses demonstrated it to have equivalent effects to mastectomy in terms of disease-free and overall survival [1–8]. Radiotherapy is regarded as an essential component of breast-conserving treatment because it markedly reduced local recurrence in the randomized trials [7, 9–11], and meta-analysis [12] demonstrated that radiotherapy significantly reduced not only locoregional recurrence, but also distant recurrence and breast cancer deaths. However, pathologically negative node, old age, and positive hormonal receptor status are known to reduce local recurrence rates. Therefore, randomized trials comparing radiotherapy with no radiotherapy after breast-conserving surgery have been conducted for elderly patients with node-negative and estrogen receptor-positive breast cancer treated using adjuvant hormonal agents [14]. Radiotherapy reduced the risk of IBTR, but it did not impact distant recurrence or overall survival of early breast cancer treated by breast-conserving surgery and tamoxifen in elderly women (70 years or older) in CALGB 9343. The IBTR rate was 9% at 10 years in the no radiotherapy group. McCormick et al. reported that 88% of women aged 70 years and older with stage I, estrogen receptor-positive breast cancer received radiotherapy after breast-conserving surgery, even after the National Comprehensive Cancer Network (NCCN) guidelines for elderly breast cancer patients stated the omission of radiotherapy after breast-conserving surgery in women meeting the CALGB 9343 entry criteria who were prescribed hormonal agents in 2009 [15]. Two other groups also reported low IBTR rates in 65-year-old or older breast cancer patients [16, 17]. One study is a randomized trial of PRIME II. This trial randomly assigned women with hormone receptor-positive and node-negative breast cancer of 3 cm or less and negative excision margins (1 mm or more) into either whole-breast radiotherapy or no radiotherapy. At median follow-up of 5 years, actuarial ipsilateral breast cancer recurrence was 1.3% in women allocated whole-breast radiotherapy and 4.1% in those assigned no radiotherapy. The other study demonstrated a very low rate of IBTR after breast-conserving surgery without radiotherapy [17]. This was a single arm study. They reported an IBTR rate of 1.2% at 5 years in 601 participants with hormonal agents. They used the criteria of T1 tumor, node-negative, ER-positive, and Elston–Ellis histological grade 1 or 2. However, they used sector resection of the tumor, the area of which was larger than that of local excision that we used.

Our first study, WORTH 1, was started to identify a population with breast cancer who had a low risk of IBTR after breast-conserving surgery without radiotherapy shortly after the first reports of the study of Fyles et al. [18] and the CALGB 9343 study [19] in 2001. We included the “5-mm-thick slice and 5-mm free-margin” method to reduce the IBTR rate, and “no lymphatic invasion around the tumor”, which was a possible factor to avoid inflammatory-type IBTR, in the eligibility criteria. After WORTH 1 was started, the IBTR rate was low. Therefore, we started the second study, WORTH 2, with the same eligibility criteria as WORTH 1 to ensure low IBTR rates.

The 5-year IBTR-free rates were 94.8%, 98.4%, and 97.0% in WORTH 1, WORTH 2, and in combination, respectively. The rate was lower in WORTH 1 than in WORTH 2 even though we used the same eligibility criteria. One reason for the difference is the hormonal agents used. The most frequently used agent was tamoxifen in WORTH 1, but it was anastrozole in WORTH 2. Aromatase inhibitors, including anastrozole, are more effective at reducing hormone receptor-positive breast cancer recurrence [20]. The second possible reason was the difference in PR-negative rates. Thirty-seven of 123 patients were PR negative in WORTH 1, whereas 27 of 198 were PR negative in WORTH 2 (30.1% vs. 13.6%, *P* = 0.0003 by chi-square test). The last possible factor was MRI use before the surgery. Recently, we often use MRI to make sure that multifocality or multicentricity is not present before the surgery in Japan, but we did not collect the data of MRI use.

 The “5-mm interval slice and 5-mm margin-free” method were used in both WORTH 1 and 2. This method had been used to judge pathologically negative margins in Japan. This method is labor- and time-consuming, but effective in reducing the IBTR rate because the combined 5-year IBTR-free rate was 97.0%, which was high.

Two factors, age at the time of surgery and PR status, were identified as independent prognostic factors for the IBTR rate in this combined analysis. The IBTR-free rate of patients aged 65 years or older at the time of surgery with PR-positive tumors was 98.4% at both 5 and 10 years. Wickberg et al. reported that luminal A tumors have a lower IBTR rate than luminal B tumors after breast-conserving surgery with or without radiotherapy [21]. Based on this favorable rate, we concluded that patients aged 65 years or older with both estrogen and progesterone receptor-positive breast cancer of 3 cm or smaller by palpation, who underwent breast-conserving surgery, had histologically negative margins judged by the “5-mm interval slice and 5-mm margin-free” method and no lymphatic invasion around the tumor do not need radiotherapy.

The strong points of our studies were the relatively large sample size and prospective design, but there were some limitations. First, our studies were not randomized. Therefore, we cannot measure the effects of radiotherapy. However, this was not the purpose of our studies. Second, it is unclear whether our “5-mm interval slice and 5-mm margin-free” method is effective for reducing the IBTR rate. ASCO recommends the criteria of no tumor cells on inked margins for histologically negative margins [22]. However, ASCO guidelines only consider patients who will receive radiotherapy. Our “5-mm interval slice and 5-mm margin-free” method may be useful to select patients who do not need radiotherapy. The previously mentioned study reported a low IBTR rate of 1.2% at 5 years [17]. They enrolled patients with PR-positive breast cancer, comprising 89.1% of the cohort and used sector resection of the breast. The area of sector resection is larger than that of local excision which we used. So, we believe that our “5-mm interval slice and 5-mm margin-free” method was effective to reduce IBTR in local excision. Third, the IBTR rate of WORTH2 was lower than that of WORTH1. The reasons for this were thought to be the higher rates of PR positivity and use of aromatase inhibitors in WORTH2, but there might have been a higher rate of the use of MRI before surgery in WORTH2. MRI might have detected the multiple cancers in the breast. We did not collect the data on MRI use. Therefore, this might have been one of the reasons. Lastly, the percentages of ER and/or PR-positive tumor cells in the tumors were not obtainedd. However, patients with higher percentages of these receptors may have been enrolled in the studies.

## Conclusions

Our “5-mm-thick slice and 5-mm margin-free” method may be effective for selecting patients who can be treated by breast-conserving surgery and hormone therapy without radiotherapy. Patients who fulfill our eligibility criteria for WORTH 1 and 2 and HER2 negative, and aged 65 years or older at the time of surgery with PR-positive tumors, may not need radiotherapy if they receive hormonal agents.

## Data Availability

Please e-mail Ohsumi S.

## Code availability

StatView 5, SAS Institute Inc.
